# Effects of pharmacological neurotrophin receptor inhibition on bladder function in female mice with cyclophosphamide-induced cystitis

**DOI:** 10.3389/fruro.2022.1037511

**Published:** 2022-11-08

**Authors:** Harrison W. Hsiang, Beatrice M. Girard, Lexi Ratkovits, Susan E. Campbell, Margaret A. Vizzard

**Affiliations:** The Larner College of Medicine, Department of Neurological Sciences, The University of Vermont, Burlington, VT, United States

**Keywords:** interstitial cystitis (IC)/bladder pain syndrome (BPS), nerve growth factor, brain-derived neurotrophic factor (BDNF), urinary bladder, cyclophosphamide, p75, TrkA, TrkB

## Abstract

Interstitial cystitis/bladder pain syndrome is a chronic inflammatory pelvic pain syndrome of unknown etiology characterized by a number of lower urinary tract symptoms, including increased urinary urgency and frequency, bladder discomfort, decreased bladder capacity, and pelvic pain. While its etiology remains unknown, a large body of evidence suggests a role for changes in neurotrophin signaling, particularly that of nerve growth factor (NGF) and brain-derived neurotrophic factor (BDNF). Here, we evaluated the effects of pharmacological inhibition of the NGF receptor TrkA, BDNF receptor TrkB, and pan-neurotrophin receptor p75^NTR^ on bladder function in acute (4-hour) and chronic (8-day) mouse models of cyclophosphamide (CYP)-induced cystitis. TrkA inhibition *via* ARRY-954 significantly increased intermicturition interval and bladder capacity in control and acute and chronic CYP-treatment conditions. TrkB inhibition *via* ANA-12 significantly increased intermicturition interval and bladder capacity in acute, but not chronic, CYP-treatment conditions. Interestingly, intermicturition interval and bladder capacity significantly increased following p75^NTR^ inhibition *via* LM11A-31 in the acute CYP-treatment condition, but decreased in the chronic condition, potentially due to compensatory changes in neurotrophin signaling or increased urothelial barrier dysfunction in the chronic condition. Our findings demonstrate that these receptors represent additional potent therapeutic targets in mice with cystitis and may be useful in the treatment of interstitial cystitis and other inflammatory disorders of the bladder.

## Introduction

Interstitial cystitis/bladder pain syndrome (IC/BPS) is a chronic inflammatory pelvic pain syndrome of unknown etiology characterized by a number of lower urinary tract (LUT) symptoms, including increased urinary urgency and frequency, bladder discomfort, decreased bladder capacity, and pelvic pain. It is currently thought that a positive feedback loop of urinary bladder inflammation and afferent hypersensitization underlies IC/BPS. Inflammation increases excitability of bladder afferents, which in turn release inflammatory neuropeptides, growth factors, cytokines, and chemokines throughout the micturition pathway, leading to altered bladder function (1, 2). There currently exists no effective therapy for IC/BPS and the disease exacts a tremendous financial burden on both individuals and the economy as a whole (3).

While the etiology of IC/BPS remains unknown (4), a large body of evidence suggests a role for changes in neurotrophin signaling, particularly that of nerve growth factor (NGF) and brain-derived neurotrophic factor (BDNF) (5–8). Both NGF and BDNF are well-implicated in the LUT symptoms in overactive bladder and IC/BPS. Previous studies have shown upregulation of NGF at sites of tissue inflammation (9, 10), changes in its expression in the urine and bladders of both rodents and humans with cystitis (11–14), and changes in bladder function consistent with cystitis as a consequence of its urothelial overexpression (15, 16) or administration to the bladder (17, 18). BDNF is upregulated throughout the micturition pathway in both humans and rodents with cystitis (19–21) as a consequence of increased NGF synthesis (22, 23) and its reduction is associated with subjective improvement in IC/BPS patients undergoing treatment (20). Pharmacological disruptions of both NGF and BDNF in models of bladder inflammation have given complementary results, improving bladder function (15, 24, 25). However, anti-NGF treatments for a variety of pain conditions have been halted due to severe side effects (26, 27). Thus, there is a clear need for additional therapeutic targets.

Neurotrophin signaling can alternatively be targeted at the receptor level. NGF activates two distinct receptors, the prosurvival high-affinity tyrosine receptor kinase A (TrkA) receptor and the pro-apoptotic low-affinity pan-neurotrophin receptor p75^NTR^ (28–31). Under certain ratios of coexpression, p75^NTR^ increases TrkA affinity for NGF, thus modulating NGF signaling (32). Most BDNF interactions are mediated through the high-affinity TrkB receptor, although it can also activate p75^NTR^ (28–30). Here, we show the effects of pharmacological inhibition of p75^NTR^, TrkA, and TrkB on bladder function in a mouse model of cyclophosphamide (CYP)-induced cystitis. Our findings demonstrate that these receptors represent additional potent therapeutic targets in mice with cystitis and may be useful in the treatment of IC/BPS and LUT symptoms in other inflammatory disorders of the bladder.

## Methods

### Animals

Female C57BL/6 wildtype (WT) mice used in this study were bred locally at the Larner College of Medicine at the University of Vermont. The litters were of normal size, weight, and activity (feeding, drinking, behaviors). The average litter size was 6-8 mice. Mouse litters were undisturbed and not manipulated. Mice from different litters were assigned with simple randomization by distributing experimental groups across multiple cages and litters. The UVM Institutional Animal Care and Use Committee approved all experimental protocols involving animal usage (IACUC #X9-020). Animal Care was under the supervision of the UVM Office of Animal Care Management in accordance with the Association for Assessment and Accreditation of Laboratory Animal Care (AAALAC) and National Institutes of Health (NIH) guidelines. Estrous cycle status was not determined in female mice before use. All efforts were made to minimize the potential for animal pain, stress, or distress. Separate groups of littermate WT were used in the following experiments.

### Bladder catheter implantation

Female adult WT mice (3-4 months) were anesthetized with 2-3% isoflurane in oxygen. A mid-abdominal incision was made, allowing access to the urinary bladder, into the dome of which a small hole was made and flared-tip PE-50 tubing inserted. The tubing was run subcutaneously to the nape of the neck and coiled. A small incision was made and a wax-sealed port anchored *via* stitching to the nape of the neck, allowing access to the tubing. All incisions were stitched up and the mice given a three-day recovery period. During this time, the mice also received a postoperative analgesic, carprofen, administered subcutaneously (s.c.; 0.1 mL/10g) for 48 hours following surgery. Induction of cystitis *via* CYP began following this period. Cystometry was conducted following appropriate incubation periods for both acute and chronic CYP treatments.

### CYP-induced cystitis

Mice (N = 5-8, per treatment group) received cyclophosphamide (CYP) intraperitoneally (i.p.) to create acute (4-hour incubation, 200 mg/kg) or chronic (75 mg/kg every third day for a total of three injections) treatment groups (14, 33, 34). CYP is metabolized to acrolein, an irritant then expelled in the urine. (35) Injections were performed under 3% isoflurane anesthesia. The control group received no CYP treatment.

### Conscious, open-outlet cystometry

Following the recovery period, tubing was exteriorized and the mice were placed, unrestrained, in a wire-bottomed recording cage and the catheter connected, *via* a T-tube, to a pressure transducer (Crass Model PT300, West Warwick RI, USA) and microinjection pump (Harvard Apparatus 22, South Natick MA, USA). Room temperature saline (0.9%) was infused into the bladder (25 μL/minute). The following urodynamic parameters were recorded using a Small Animal Cystometry Lab Station (MED Associates, St Albans VT, USA): bladder pressure (threshold, maximum, minimum, and average), infused volume (IV; the volume of saline infused into the bladder since the last void), and intermicturition interval (IMI). Bladder capacity is defined as the IV necessary to elicit a micturition event. At least six reproducible micturition cycles were recorded per mouse following a 15-minute acclimation period (33, 35). Mice in these studies had residual volume of less than 5 μL, meaning infused volume and void volume were similar. Testing was conducted at similar times of day to mitigate any impact of circadian rhythm. Bladder pressure measurements between mice displayed high variability and no statistical differences were observed between groups in the present study.

### Intravesical administration of pharmacological inhibitors

Following initial cystometry, each treatment group received intravesical delivery of: 30 mg/kg ARRY-952 selective TrkA inhibitor in 20% Captisol vehicle (AR; Pfizer, New York NY, USA), 100 mg/kg selective p75^NTR^ inhibitor LM11A-31 (LM; Ricerca Biosciences, Painesville OH, USA) in sterile, injectable water, 100mg/kg selective TrkB inhibitor ANA-12 (ANA; MedChem Express, Monmouth Junction NJ, USA) in 10% dimethyl sulfoxide (DMSO) over thirty minutes. 0.5mL of inhibitor was delivered to the bladder over a 30-minute period for each treatment group. Vehicle controls found no effects of the Captisol vehicle alone (Figure 3). Subsequently, the mice then underwent another session of cystometry, allowing each mouse to serve as its own baseline pre- and post-administration of inhibitors. Following the final cystometry session, mice were deeply anesthetized with 5% isoflurane in oxygen and euthanized *via* thoracotomy.

### Exclusion criteria

Mice were withdrawn from the study due to postoperative complications or pain, lethargy, or distress that could not be addressed with postoperative analgesics. Some cystometry recordings could not be used due to artifacts from excessive behavioral movements (such as grooming and chewed tubing); in CYP-treatment conditions, mice exhibiting neither bladder functional impairment nor signs of bladder inflammation (blistering of the urothelium, edema, and sloughing of urothelial cells) on visual inspection after euthanasia were excluded as well. Approximately 10% of mice were removed from the study.

### Cystometry analysis

Cystometry traces were analyzed offline using MED-CMG software (Med Associates, St Albans VT, USA) and R. Values of functional bladder parameters were averaged before and after treatment for each mouse and averaged for each treatment group. Results were statistically analyzed using Welch’s paired t-tests and one-way analysis of variance (ANOVA) with Tukey’s honestly significant difference (HSD) *post-hoc* analysis where appropriate. *P*-values less than or equal to 0.05 were considered statistically significant. Asterisks (*, **, ***) indicate statistical differences at the *p* ≥ 0.05, 0.01, and 0.001 levels. Data is presented as boxplots with range and individual slope graphs. Boxplots display the median, interquartile range, maximum, minimum, and outliers (indicated with dots). The range is indicated by the corresponding bolded portions of the y-axis. Slope graphs indicate the change of individual mice before and after treatment. Red dotted lines indicate the change in mean values for each condition before and after treatment.

## Results

### Both acute (4-hour) and chronic (8-day) CYP-treatment reduced intermicturition interval and bladder capacity

**FIGURE 1:**
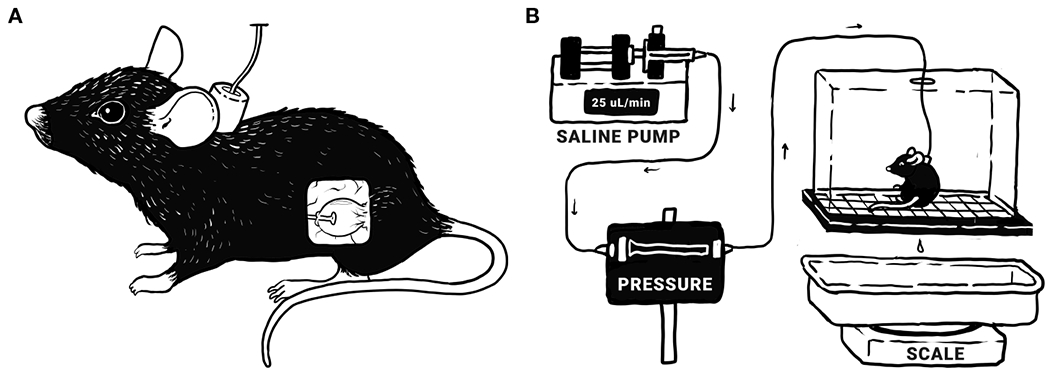
Bladder function was assessed *via* conscious, open-outlet cystometry. **(A)** Tubing is surgically implanted in the bladder and run subcutaneously to a port at the nape of the neck, allowing direct infusion into the bladder. **(B)** During cystometry, the mouse is placed in a wire-bottomed recording chamber. Saline is infused into the bladder at a constant rate of 25 μL/minute, allowing the measurement of various urodynamic parameters: bladder pressure (threshold, maximum, minimum, and average), infused volume (IV; the volume of saline infused into the bladder since the last void), and intermicturition interval (IMI; time between voids). Original illustrations by Harrison Hsiang.

**FIGURE 2:**
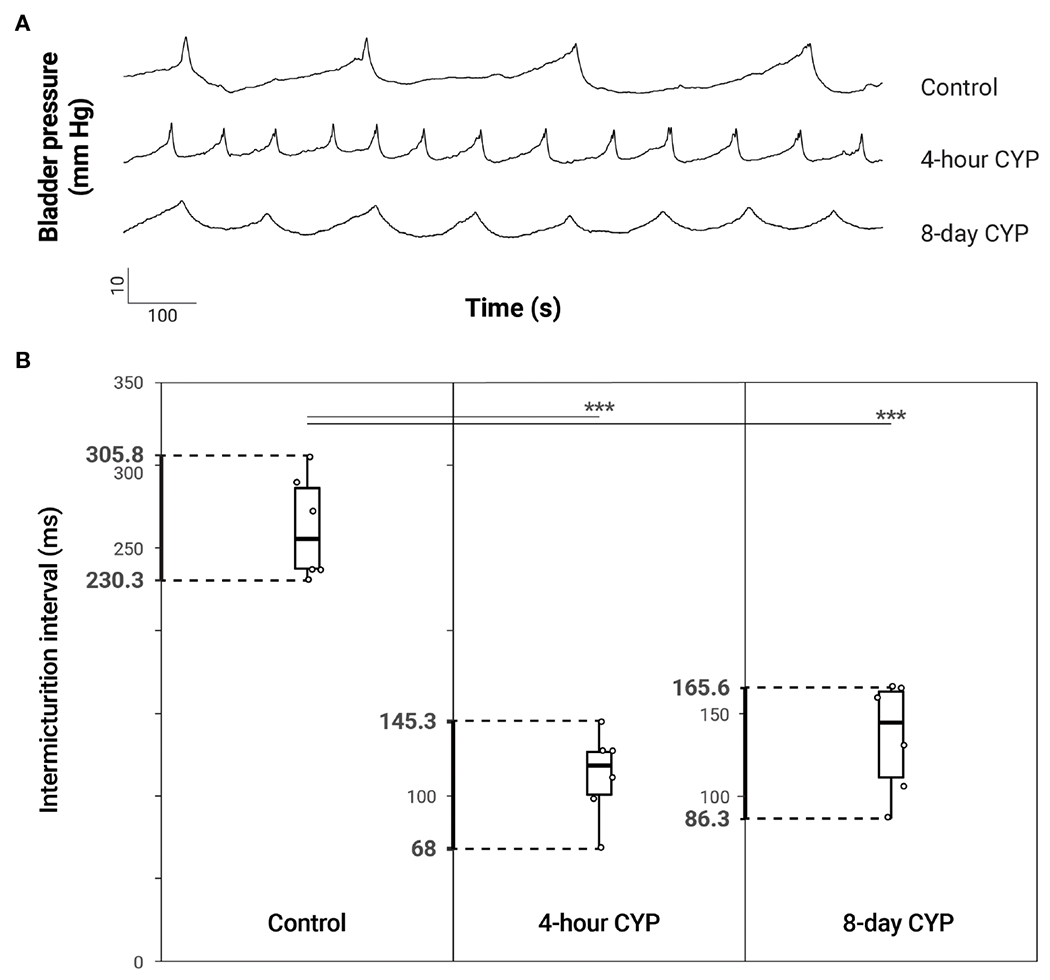
Increased voiding frequency following acute (4-hour) and chronic (8-day) CYP-treatment. **(A)** Representative traces of bladder pressure (mm Hg) over time (seconds) for control, acute and chronic CYP-treatment during constant intravesical infusion of saline. Bladder pressure increases with filling, spiking when the bladder contracts during micturition. Intermicturition interval is visibly decreased (increased frequency) in acute and chronic CYP-treatment conditions. **(B)** Both acute and chronic CYP-treatment increased voiding frequency. Intermicturition interval and infused volume were significantly reduced in acute (*p* = 0.0000014) and chronic (*p* = 0.0000098) CYP-treatment conditions. Circles mark the individual data points for each condition. *N* = 6 for all.

Bladder function was assessed through conscious, open-outlet cystometry (Figure 1). As previously demonstrated (14, 33, 34), intermicturition interval (IMI) and infused volume (IV) were statistically significantly reduced in both the acute (4-hour, 200 mg/kg i.p.) and chronic (8-day, 75 mg/kg i.p.) cyclophosphamide (CYP)-treatment conditions when compared to the control conditions (Figure 2 and Table 1). One-way ANOVA revealed a statistical difference in IMI by condition (F(2,15) = 40.95, *p* = 8.38x10^−7^) and Tukey’s HSD Test for multiple comparisons revealed that the mean IMI value was significantly different between control and acute CYP (*p* = 0.0000014) and control and chronic CYP conditions (*p* = 0.0000098). One-way ANOVA also revealed a statistical difference in IVby condition (F(2,15)= 40.83, *p* = 8.54x10^−7^) and Tukey’s HSD Test for multiple comparisons revealed that the mean IV value was significantly different between control and acute CYP (*p* = 0.0000014) and control and chronic CYP conditions (*p* = 0.00001; Table 1).

### TrkA inhibition *via* AR reduced void frequency and increased bladder capacity in both the acute and chronic CYP-induced cystitis models

**FIGURE 3:**
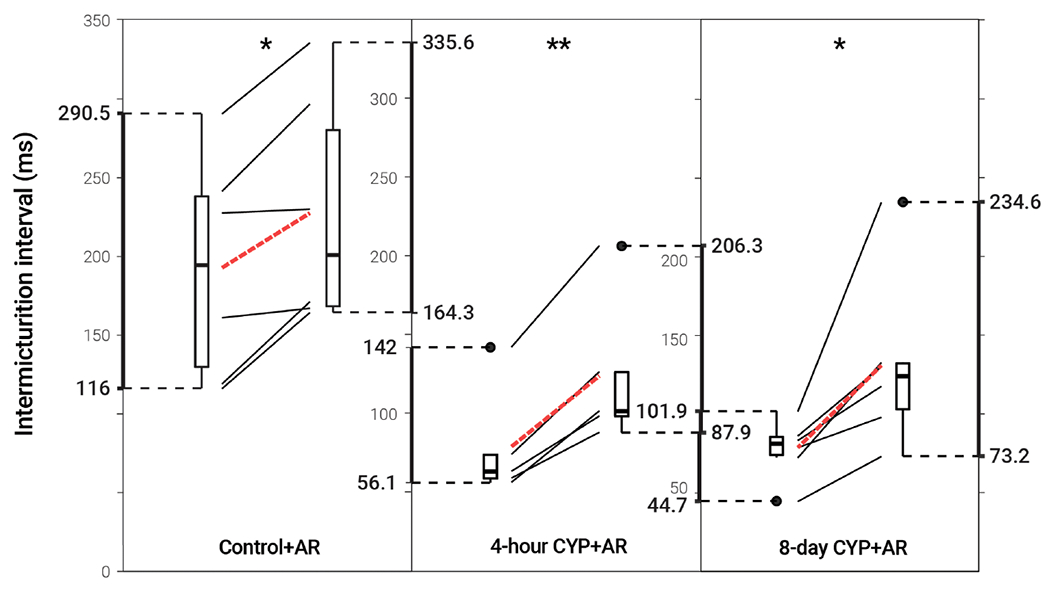
Decreased voiding frequency following intravesical treatment with AR. Intermicturition interval was statistically increased following intravesical administration of AR in control (1.18-fold, *p* = 0.01622, *N* = 6) and acute (4-hour; 1.56-fold, *p* = 0.001935, *N* = 5) and chronic (8-day; 1.68-fold, *p* = 0.02549, *N* = 6) CYP-treated mice. Slope graphs indicate the change of individual mice before and after treatment. Red dotted lines indicate the change in mean values for each condition before and after treatment.

**FIGURE 4:**
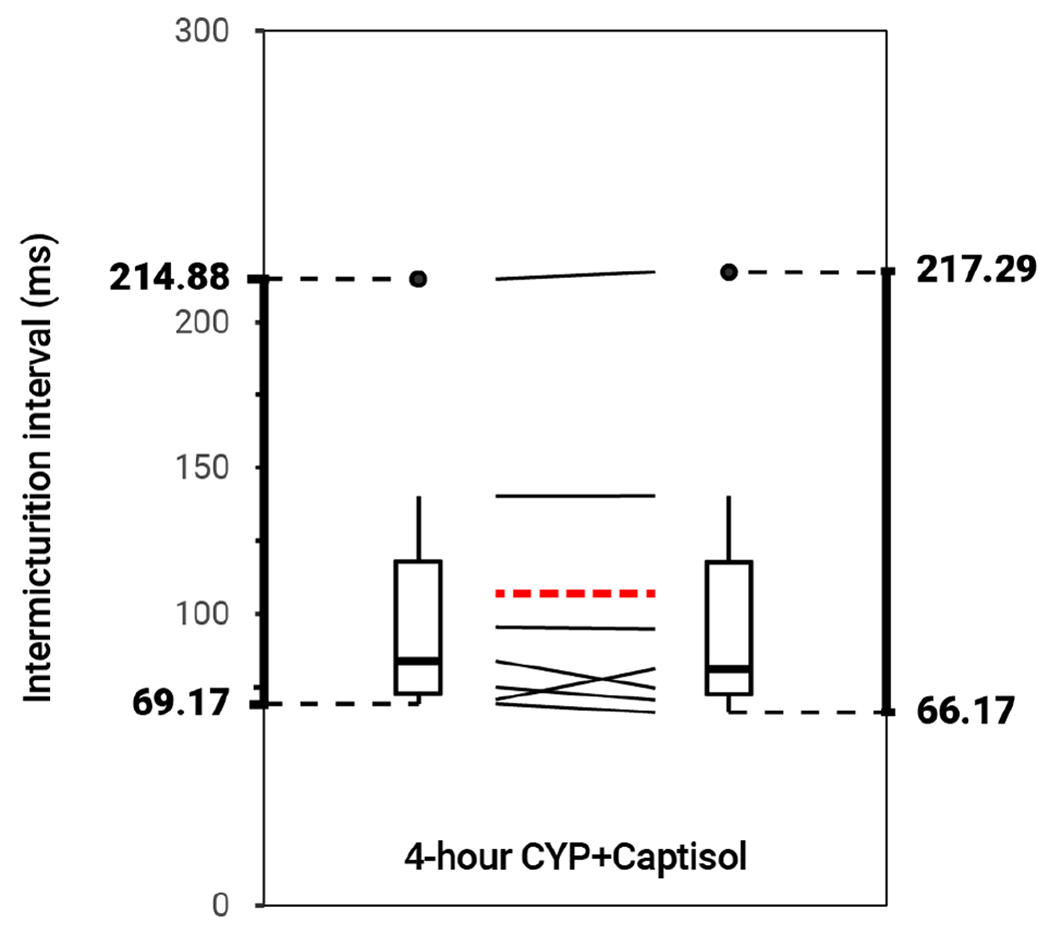
No effect on bladder function following intravesical treatment with 20% Captisol (*p* > 0.05, *N* = 6). Slope graphs indicate the change of individual mice before and after treatment. Red dotted lines indicate the change in mean values for each condition before and after treatment.

Intravesical administration of ARRY-954 (AR) statistically increased IMI and IV in control (*p* = 0.01622; *p* = 0.01633) and acute (*p* = 0.001935; *p* = 0.01954) and chronic (*p* = 0.02549; *p* = 0.02548) CYP-induced cystitis conditions. IMI interval increased 1.18-fold in control mice, 1.56-fold in acute CYP-treated mice, and 1.68-fold in chronic CYP-treated mice (Figure 3 and Table 2). AR was delivered intravesically in 20% Captisol. Vehicle controls found no effect of vehicle alone (*p* > 0.05; Figure 4).

### p75^NTR^ inhibition *via* LM reduced void frequency and increased bladder capacity in acute CYP-treated mice, but reduced function in chronic CYP-treated mice

**FIGURE 5:**
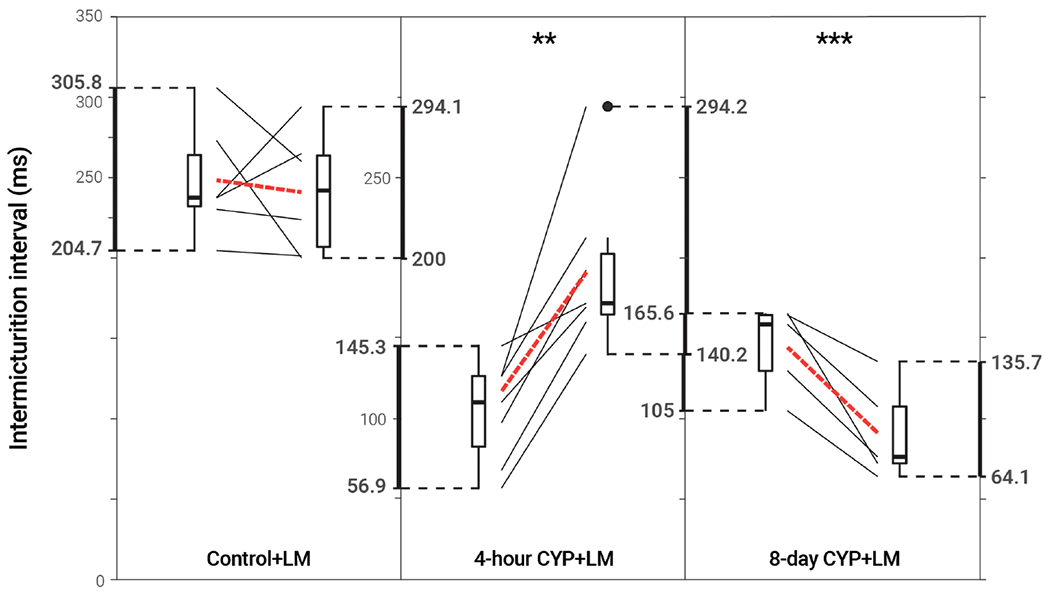
Decreased voiding frequency following intravesical treatment with LM in acute CYP-treatment conditions. Intermicturition interval was statistically increased following intravesical administration of LM in acute (4-hour) CYP-treated mice (1.83-fold, *p* = 0.001702, *N* = 7). Intermicturition interval was statistically decreased following intravesical administration of LM in chronic (8-day) CYP-treated mice (0.63-fold, *p* = 0.007845, *N* = 5). There was no statistical difference before and after LM treatment in the control condition (*p* > 0.05; *N* = 6). Slope graphs indicate the change of individual mice before and after treatment. Red dotted lines indicate the change in mean values for each condition before and after treatment.

Intravesical administration of LM11A-31 (LM) statistically increased IMI (*p* = 0.001702) and IV (*p* = 0.001676) in acute (4-hour) CYP-treated mice, a 1.83-fold increase. However, LM administration statistically decreased IMI (*p* = 0.007845) and IV (*p* = 0.007725) in chronic (8-day) CYP-treated mice 0.63-fold. No change was observed following treatment in the control condition (*p* > 0.05; Figure 5 and Table 3).

### TrkB inhibition *via* ANA reduced void frequency and increased bladder capacity in acute but not chronic CYP-treated mice

**FIGURE 6:**
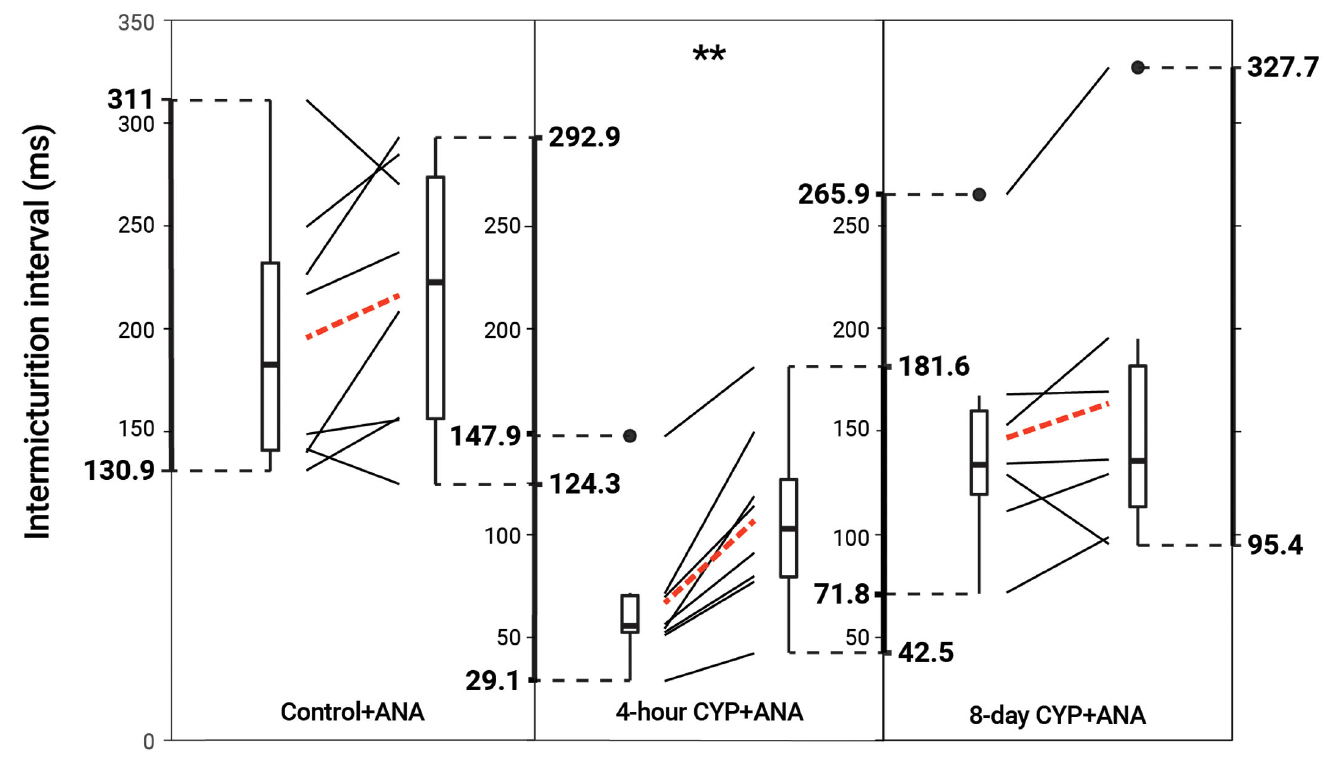
Decreased voiding frequency following intravesical treatment with ANA. Intermicturition interval was statistically increased following intravesical administration of ANA in acute (4-hour) CYP-treated mice (1.6-fold, *p* = 0.001114, *N* = 8). There was no statistical difference following treatment in the control and chronic (8-day) CYP-treatment conditions (*p* > 0.05, *N* = 8, 7). Slope graphs indicate the change of individual mice before and after treatment. Red dotted lines indicate the change in mean values for each condition before and after treatment.

Intravesical administration of ANA statistically increased IMI (*p* = 0.001114) and IV (*p* = 0.001113) in the acute CYP-treatment condition. No change was found in the control and chronic CYP-treatment conditions (*p* > 0.05; Figure 6 and Table 4).

## Discussion

This study demonstrates the different effects of p75^NTR^, TrkA, and TrkB inhibition on bladder function in control and acute or chronic CYP-treated mice.

TrkA inhibition *via* intravesical administration of AR was associated with improved bladder function, with statistical increases in both the intermicturition interval (IMI) and infused volume (IV), in control and both acute (4-hour) and chronic (8-day) CYP-treatment conditions. Unsurprisingly, accumulating evidence suggests that NGF actions in cystitis are primarily TrkA-mediated. Its sequestration has prevented the development of hyperalgesia and reduced cystitis-associated changes in voiding frequency in CYP-treated animals (16, 24, 36). A previous study has also shown functional improvement following treatment with pan-Trk inhibitor K252A in rats with CYP-induced cystitis (15). TrkA and TrkB immunoreactivity and phosphorylation are increased in the urinary bladder and its afferents following bladder inflammation (21, 37). TrkA is also implicated in the expression of the nociceptive TRPV1 receptor (38), known to be upregulated in cystitis (39, 40), as well as in the development of mechanical and thermal hyperalgesia (41–46), all of which are associated with cystitis. TrkA inhibition increased IMI and IV in the control condition, suggesting that NGF-TrkA signaling may modulate bladder function even under noninflammatory conditions.

p75^NTR^ inhibition *via* intravesical administration of LM also improved bladder function in the acute CYP-treatment condition. Given that NGF actions during cystitis may be primarily TrkA-mediated, this finding is consistent with the understanding that p75^NTR^ modulates NGF-TrkA actions when the two receptors are coexpressed, potentially even when activated by immature proneurotrophins (30, 32). In rats with thermal hyperalgesia, the magnitude of the acute response is TrkA-mediated while the duration of the response is p75^NTR^-mediated. (47) However, p75^NTR^ can also enact pro-apoptotic and growth-limiting actions independently of TrkA (28, 29, 47). p75^NTR^ and its downstream effectors are essential for the development of NGF-mediated mechanical hyperalgesia in the rat hindpaw (43). NGF-p75^NTR^ signaling–mediated changes in ceramide (via the sphingomyelin cycle) have been demonstrated to occur independently of TrkA in cell culture (48). NGF binding to p75^NTR^-sortilin complexes reduces growth (47). Additionally, p75^NTR^ activates Jun kinase (JNK) (28), which influences a number of cystitis-relevant cellular functions including apoptosis, inflammation, cytokine production, and cellular differentiation and proliferation. In thermal hyperalgesia, it seems the magnitude of the acute response is TrkA-mediated while the duration of the response is p75^NTR^-mediated (46). These studies, alongside our demonstration that p75^NTR^ inhibition improved bladder function in CYP-treated mice, suggests that p75^NTR^ may be a potent modulator of NGF-TrkA signaling–mediated bladder dysfunction and peripheral sensitization in cystitis.

Interestingly, while p75^NTR^ inhibition improved bladder function in the acute CYP-treatment condition, it was associated with reduced bladder function in the chronic condition, with both IMI and IV decreasing after treatment. This may result from compensatory changes in protein expression under chronic inflammation conditions. Girard et al. (16) observed reduced expression of TrkA and TrkB and increased expression of p75^NTR^ in urothelium-specific NGF-verexpressing mice, which the authors postulated may represent compensatory, concomitant changes to reduce urinary frequency. These compensatory changes in protein expression likely disrupt p75^NTR^-mediated facilitation of NGF-TrkA actions, which depend on high TrkA:p75^NTR^ coexpression (30, 32), instead allowing p75^NTR^ actions to oppose NGF-TrkA actions. p75^NTR^ inhibition under these conditions may undermine natural compensatory changes and consequently reduce bladder function. An alternative explanation may be that the effects of p75^NTR^ inhibition differ between the urothelium and regions of the bladder deep to the urothelium; Klinger et al. (49) found that PD90780, known to disrupt NGF-p75^NTR^ signaling, produces bladder overactivity in control and CYP-treated rats only when infused with protamine sulfate, which disrupts urothelial barrier function. Here, reduced bladder function following LM infusion may result from potentially increased urothelial barrier dysfunction at the chronic timepoint, allowing LM to penetrate deeper into the bladder wall. Chronic CYP-treated mice display urothelial hyperplasia without commensurate increases in mRNA expression for urothelial tight junction proteins, suggesting urothelial barrier function is indeed compromised in the chronic condition (50), which may influence the penetration rate of the inhibitors tested here.

TrkB inhibition *via* intravesical administration of ANA improved bladder function in the acute but not chronic CYP-treatment condition. This is consistent with previous studies. BDNF is well-implicated in acute inflammatory responses in the bladder. Expression of BDNF and TrkB increase throughout the micturition pathway – particularly the urothelium – in response to bladder inflammation, spinal cord injury, and bladder outlet obstruction (19–21, 37, 51, 52); furthermore, overexpression of BDNF in the bladder wall induces bladder overactivity and increases expression of nociceptive TRPV1 and TRPA1 channels and cholinergic and purinergic signaling proteins (53) and its sequestration improves bladder function and reduces expression of cFOS and phosphorylated ERK, known markers of noxious input (54–56), in rats with acute CYP-induced cystitis (19). However, there is some indication that BDNF actions in the bladder are short-lived. Oddiah et al. (12) found that BDNF expression was increased when measured two hours after turpentine-induced inflammation but not at six hours after. This is consistent with our finding that TrkB inhibition improved bladder function in the acute (4-hour) but not chronic (8-day) CYP-treatment conditions. A possible explanation is increased retrograde transport of BDNF to the dorsal root ganglia and spinal dorsal horn at later time points in cystitis, contributing to central windup. BDNF is upregulated in the spinal dorsal horn following CYP treatment, promoting astrocyte and microglia activation and, in turn, increased inflammation and mechanical allodynia (57); delivery of exogenous BDNF intrathecally reproduces symptoms of bladder hyperactivity and pain, and its blockade or sequestration reduces them. NGF is known increase peripheral and spinal levels of BDNF, which is then transported retrogradely to central and peripheral nerve terminals (58). BDNF notably increases sensory neuron excitability through p75^NTR^ (59), and its role in central sensitization is thought to underlie many pain conditions (60). Notably, BDNF actions in the spinal cord only persist 10-20 minutes after administration (61), likely due to receptor internalization and diffusion or degradation of BDNF (22, 62). Here, due to the intravesical delivery of inhibitors, it is likely treatment primarily affected receptors expressed in the bladder, however, a number of studies have demonstrated effects of inhibition of various neurotrophin signaling–related proteins at the DRG level as well (41, 63, 64). These studies implicate neurotrophin signaling at the DRG level in the modulation of pain sensation in addition to its functional effects at the level of the bladder, demonstrated here. Future studies addressing intrathecal or systemic administration of agents will help to evaluate contributions from receptors expressed in the DRG and more widely throughout the body.

There are several limitations to the present study. First, the effects of these inhibitors were only evaluated in a mouse model of cystitis. CYP-induced cystitis is a reliable, well-validated, and extensively characterized model that reproduces the neurochemical and functional changes and localized bladder inflammation symptoms of IC/BPS (50, 65–67); of particular relevance, mice appear to be more robust to systemic CYP treatment than rats, exhibiting detrusor overactivity, increased urinary frequency, and lower abdominal hyperalgesia without dramatic alterations in physiological state, body temperature, or weight (68), more closely modeling the chronic nature of IC/BPS. However, we acknowledge that the 8-day model is more akin to repeat acute inductions rather than a true chronic condition; there is currently a paucity of chronic models for cystitis. Nonetheless, all animal models have limitations that may limit therapeutic translation of these results. Cross-validation in a number of available alternative animal models such as other irritant-induced cystitis models, stress models, and naturally occurring cystitis in cats (67, 69), will provide additional evidence as to the role of neurotrophin signaling in cystitis and other inflammatory disorders of the bladder. Additionally, these studies were conducted solely in female mice, which may leave sex differences unaccounted for. IC/BPS disproportionately affects women over men at a rate of 10:1 (70, 71); however, chronic prostatitis/pelvic pain syndrome in men has considerable clinical overlap with IC/BPS with recent suggestion that male IC/BPS may be under- and misdiagnosed (72). Future studies will benefit from incorporating male subjects. Finally, we did not investigate the effects of the various inhibitors in combination, which may reveal synergistic effects and allow for lower doses of each individual inhibitor to achieve the same effect.

The present study demonstrates that p75^NTR^, TrkA, and TrkB are potent therapeutic targets in the treatment of cystitis. Pharmacological inhibition of all three receptors was associated with strong improvement in LUT symptoms in the acute cystitis condition. TrkA inhibition improved bladder function in the chronic cystitis condition as well, while findings resulting from p75^NTR^ and TrkB inhibition provide further insight into the roles of NGF and BDNF signaling in sustained conditions of bladder inflammation.

## Figures and Tables

**TABLE 1:** Mean ± SEM values for intermicturition interval and infused volume for control and acute (4-hour) and chronic (8-day) CYP-treatment conditions.

Condition	Intermicturition interval (ms)	Infused volume (mL)
Control	262.48 ± 12.97	109.51 ± 5.42
4-hour CYP	112.38 ± 11.08***	46.99 ± 4.61***
8-day CYP	135.00 ± 55.12***	56.46 ± 23.05***

Intermicturition interval and infused volume were significantly reduced in acute (p = 0.0000014) and chronic (p = 0.0000098) CYP-treatment conditions. N = 6 for all.

**TABLE 2:** Mean ± SEM values for intermicturition interval and infused volume before and after AR treatment.

	Intermicturition Interval (ms)		Infused Volume (mL)	
Condition	Before	After	Before	After
Control	192.56 ± 29.13	227.45 ± 30.17*	80.41 ± 12.14	94.93 ± 12.57*
4-hour CYP	78.76 ± 14.69	124.03 ± 19.65**	32.96 ± 6.12	51.80 ± 8.18**
8-day CYP	77.94 ± 7.76	131.17 ± 22.61*	32.65 ± 3.24	54.80 ± 9.42*

Intermicturition interval and infused volume were statistically increased following intravesical administration of AR in control (p = 0.01622, p = 0.01633, N = 6) and acute (4-hour; p = 0.01954, p = 0.001935, N = 5) and chronic (8-day; p = 0.02549, p = 0.02548, N = 6) CYP-treated mice.

**TABLE 3:** Mean ± SEM values for intermicturition interval and infused volume before and after LM treatment.

	Intermicturition Interval (ms)		Infused Volume (mL)	
Condition	Before	After	Before	After
Control	248.18 ± 14.58	240.73 ± 15.61	103.53 ± 6.08	100.45 ± 6.51
4-hour CYP	104.45 ± 12.27	191.62 ± 19.19**	43.69 ± 5.11	80.03 ± 7.99**
8-day CYP	144.74 ± 11.88	91.22 ± 13.33***	60.52 ± 4.96	38.16 ± 5.55***

Intermicturition interval and infused volume were statistically increased following intravesical administration of LM in acute (4-hour) CYP-treated mice (p = 0.001702, p = 0.001676, N = 7). Intermicturition interval was statistically decreased following intravesical administration of LM in chronic (8-day) CYP-treated mice (p = 0.007845, p = 0.007725, N = 5). There was no statistical difference before and after LM treatment in the control condition (p > 0.05; N = 6).

**TABLE 4:** Mean ± SEM values for intermicturition interval and infused volume before and after ANA treatment.

	Intermicturition Interval (ms)		Infused Volume (mL)	
Condition	Before	After	Before	After
Control	195.45 ± 23.16	216.12 ± 23.00	81.16 ± 9.64	90.22 ± 9.58
4-hour CYP	66.71 ± 12.48	107.03 ± 15.60**	27.94 ± 5.20	44.76 ± 6.50**
8-day CYP	147.71 ± 25.06	164.69 ± 32.78	61.67 ± 10.45	68.76 ± 13.65

Intermicturition interval and infused volume were significantly increased in the acute (4-hour, p = 0.001114, p = 0.001113, N = 8) but not control or chronic CYP-treatment conditions (p > 0.05, N = 8,7).

## Data Availability

The raw data supporting the conclusions of this article will be made available by the authors, without undue reservation.
